# Ubiquitination in plant nutrient utilization

**DOI:** 10.3389/fpls.2013.00452

**Published:** 2013-11-12

**Authors:** Gary Yates, Ari Sadanandom

**Affiliations:** School of Biological and Biomedical SciencesDurham University, Durham, UK

**Keywords:** ubiquitin, plants, nutrients, abiotic stress, signaling

## Abstract

Ubiquitin (Ub) is well-established as a major modifier of signaling in eukaryotes. However, the extent to which plants rely on Ub for regulating nutrient uptake is still in its infancy. The main characteristic of ubiquitination is the conjugation of Ub onto lysine residues of acceptor proteins. In most cases the targeted protein is rapidly degraded by the 26S proteasome, the major proteolysis machinery in eukaryotic cells. The Ub-proteasome system is responsible for removing most abnormal peptides and short-lived cellular regulators, which, in turn, control many processes. This allows cells to respond rapidly to intracellular signals and changing environmental conditions. This perspective will discuss how plants utilize Ub conjugation for sensing environmental nutrient levels. We will highlight recent advances in understanding how Ub aids nutrient homeostasis by affecting the trafficking of membrane bound transporters. Given the overrepresentation of genes encoding Ub-metabolizing enzymes in plants, intracellular signaling events regulated by Ub that lead to transcriptional responses due to nutrient starvation is an under explored area ripe for new discoveries. We provide new insight into how Ub based biochemical tools can be exploited to reveal new molecular components that affect nutrient signaling. The mechanistic nature of Ub signaling indicates that dominant form of any new molecular components can be readily generated and are likely shed new light on how plants cope with nutrient limiting conditions. Finally as part of future challenges in this research area we introduce the newly discovered roles of Ub-like proteins in nutrient homeostasis.

## INTRODUCTION

Our understanding of the layers of regulation that control the cell is deepening at a rapid rate. Much like how the discovery of microRNAs and epigenetics caused major rethinking of well-established gene control systems, protein modification processes are proving to have significant roles in the control of protein function. An example of this is ubiquitination, as a post-translational modifier it is well-known as a system involved in protein turnover, and to a lesser extent is known for its roles in membrane trafficking, DNA repair, chromatin remodeling, and hormone synthesis. However, recent publications have revealed that in addition to its extensive role is stress signaling, ubiquitination also has roles in nutrients utilization. We highlight the importance of the role ubiquitin (Ub) plays in plants ability to uptake and process nutrients using recent examples. It is clear that this area of research is in its infancy and much work has to be done in order to understand the extent to which ubiquitination influences this field.

## UBIQUITINATION

Ubiquitin is a small seventy-six amino acid peptide that is highly conserved throughout eukaryotes. Ub is conjugated to the target protein through linkage between a C-terminal glycine and one or more of its seven possible lysine residues. Which of the seven lysine residue forms the attachment and the topology of the subsequent Ub chain directs the fate of the protein. Polyubiquitination via lysine 48 is usually associated with proteasomal degradation and in yeast and mammalian cells mono-ubiquitination and multiubiquitination are precursors to endocytic sorting and degradation via the lysosome and vacuole (Mukhopadhyay and Riezman, 2007). Once the target and the proteasome are connected, deubiquitinating enzymes remove the poly-Ub chain from the target protein, the Ub molecules are recycled, and the target is unfolded and fed into the 26S proteasome for proteolysis (Hartmann-Petersen et al., 2003). Opposed to lysine 48, attachment at lysine 63 is linked to endosomal degradation and trafficking (Duncan et al., 2006). Ub has five other lysines which can also take part in target conjugation, however, the precise physiological implications of these lysine linkages are yet to be discovered.

There are three main steps to Ub attachment to target proteins, each requiring a different enzyme type categorized as E1, E2, and E3. The first of these, E1, is an Ub activating enzyme. At a conserved cysteine residue, E1 forms a high-energy thioester bond with a C-terminal glycine of the Ub molecule, a step that requires ATP (Hatfield et al., 1997). Ub, in its active form, is passed from the E1 to a cysteine residue in the E2 Ub-conjugation enzyme (UBC). which forms an intermediate complex. From here Ub is transferred to the lysine on the target protein, facilitated by the Ub ligase, E3. The E3 protein, of which there are two major subclasses in *Arabidopsis*; containing either a Really Interesting New Gene (RING)- or homologus to E6-AP carboxyl terminus (HECT)-domains, has the specificity to get the Ub to the appropriate target. This is achieved either by direct transfer from E2 to the substrate (RING domain E3) or by the formation of an Ub-E3 intermediate complex (HECT domain E3, Vierstra, 2009). In the *Arabidopsis* genome there are at least 16 genes encoding the Ub molecule itself, two genes for E1’s, at least 45 genes for E2’s, and over 1400 genes encode E3’s. In combination, the elements of the Ub system (UbS) can give specificity to thousands of proteins each targeted by its unique combination of UbS components. This allows very tight control of protein levels and regulation of numerous cellular processes.

Ubiquitin system mechanisms are involved in most areas of plant life from embryogenesis to senescence (Sadanandom et al., 2012). It is postulated that the UbS enables plants to respond rapidly to changes in the environmental conditions by flexible modification of key regulators of cellular physiology.

## NUTRIENTS AND UBIQUITIN

Iron is central to many cellular processes in plants including but not limited to photosynthesis, respiration, DNA and hormone synthesis (Curie and Briat, 2003). The uptake of iron by plant roots is dependent on the protein IRON-REGULATED TRANSPORTER 1 (IRT1), which acts at the surface of root-hairs in *Arabidopsis*. A lack of available iron has severe detrimental effects on plants (Briat and Lebrun, 1999). Over accumulation of iron is detrimental in large amounts and can lead to accumulation of reactive oxygen species (Briat and Lebrun, 1999), therefore tight control of IRT1 levels is essential for maintaining iron homeostasis. During iron deprivation the IRT1 transcription is up-regulated, and in *irt1 *knockout mutant lines chlorosis forms and is quickly followed by death (Vert, 2002). This shows that IRT1 is essential for iron uptake and interestingly the endocytosis processing of IRT1 is dependent upon ubiquitination. IRT1 shuttles from the plasma membrane to the *trans*-Golgi network/early endosomes (tGN-EE), and undergoes Ub mediated vacuolar degradation (Barberon et al., 2011). Mono-ubiquitination of various lysine residues on IRT1 marks the protein for vacuole sorting; mutation of these residues renders the protein unable to transport, stabilizing it at the plasma membrane (Barberon et al., 2011). This suggests that IRT1 controls iron uptake at the transcriptional level (Vert, 2002) and also post-transcriptionally via Ub. Recently a gene has been isolated that encodes an E3-ligase which facilitates IRT1 degradation. The *IRT1 DEGRADATION FACTOR 1* (IDF1) is a RING-type E3-ligase which interacts with IRT1 and when mutated, results in reduced IRT1 degradation and hence increased iron accumulation (Shin et al., 2013).

Phosphorus is a major element essential to plant life. When active, it is crucial in both dark and light reactions of photosynthesis, as well as playing a vital role in energy metabolism and carbon assimilation. Organic phosphate is abundant in soil but inaccessible to plants. Inorganic phosphate (Pi) is generally of low availability in soils, and because of this, plants have evolved mechanisms to cope with the lack of Pi. In Pi-limited conditions plants have been shown to undergo changes in the distribution and morphology of roots, increased Pi uptake, and secretion of phosphatases and nucleases which enable the mobilization of organic phosphate (Ticconi and Abel, 2004). A major response to phosphorus deprivation is the up-regulation of a microRNA; miR399 (Chiou et al., 2006). In phosphorus poor environments miR399 regulates expression of an E2 enzyme (Fujii et al., 2005). miR399 is found at six loci in the *Arabidopsis* genome, all six transcripts are up-regulated significantly when Pi levels are low. The miR399 nucleotide sequence shows strong similarity to regions of the 5′ UTR of E2 ubiquitin-conjugation enzyme (*UBC24*), making the UBC24 transcript a likely target for decay by miR399 interaction (Allen et al., 2005). This is evident as the up-regulation of miR399 results in cleavage of the UBC24 transcript causing down regulation or even silencing of the UBC24 protein. When overexpressing miR399 in plants, accumulation of phosphate is observed and results in both increased uptake and increased translocation from the roots to the shoots (Chiou et al., 2006). Therefore given that there is interaction between miR399 and the UBC24 transcript, miR399 is most likely controlling phosphorus homeostasis by regulating both the uptake and trafficking of phosphorus, via control of the UBC24 protein level. The only suggested target of UBC24 is PHOSPHATE1 (PHO1), which is involved in the loading of phosphate to the xylem (Liu et al., 2012). Interestingly, the mammalian homolog of UBC24 is Apollon (Bartke et al., 2004) which acts as an inhibitor of cell death localized to the tGN and vesicles. Apollon has been shown to monoubiquitinate substrates in the presence of an E1 enzyme alone. Taken together, it is likely that the UBC24 also functions as an E2 with specific targets found in the tGN and vesicles.

Boron is another essential nutrient for plant life but is highly toxic in large quantities (Shorrocks, 1997). Plants depend on Borate to form a crosslinking dimer for proper formation of the cell wall component pectic polysaccharide rhamnogalacturonan II, without which plants do not develop normally and are largely non-viable (O’Neill et al., 2001). The uptake of boron depends on the transporter protein BORONTRANSPORTER1 (BOR1), and the boric acid channel protein NIP5;1. NIP5;1 is found on the soil facing surface of root cells and is essential for boric acid uptake in boron limited conditions (Takano et al., 2006). BOR1 is an efflux channel and is responsible for boron transport in low boron conditions (Takano et al., 2002, 2010). The BOR1 transporter utilizes the endocytic degradation pathway in high boron conditions where the excess is trafficked to the vacuole. This sorting of the loaded BOR1 transporter is made possible by its mono-ubiquitination or di-ubiquitination at lysine 590 and this is induced by high boron conditions. Mutation of BOR1 at lysine 590 abolishes both the ubiquitination and the degradation via the endocytic pathway (Takano et al., 2010). However, although ubiquitination is necessary for the sorting into multivesicular bodies, it is not required for BOR1 endocytosis, this process is very similar to the sorting of epidermal growth factor receptors (EGFR) in mammals (Huang et al., 2007). The E3 ligase responsible for targeting BOR1 is not yet known (Kasai et al., 2011). However, the EGFR undergoes ubiquitination by a WW (has two highly conserved tryptophans) containing domain E3 ligase which is homologous to HECT-type E3 ligase in *Arabidopsis *(Kraft et al., 2005).

There are similarities in the way BOR1 and IRT1 exploit the endocytic pathway for regulating their respective minerals, however, there is no current examples of plasma membrane transport proteins that involve processing by HECT-type E3 ligases (Mulet et al., 2013). However, it is probable, given the example of the Apollon, that there may be E2 enzymes involved in processing these membrane transporters without the need for a separate E3.

Plants need nitrogen in high abundance, as it is a building block for proteins and nucleic acids. Nitrogen limiting conditions are widespread and as such plants have evolved a range of adaptive responses to cope with the low availability. Amino acids are the major source of nitrogen transported in the xylem and phloem and they provide an important link between the nitrogen status of the roots and the rest of the plant (Paungfoo-Lonhienne et al., 2008). Amino acids can impede nitrogen uptake by roots and may play a major role in the regulation of transporters, providing part of a feedback system in sensing nitrogen (Miller et al., 2007). The import and export of amino acids in and out of the plant cells involve various membrane proteins and proton-gradient dependant transporters. The importing mechanisms are well-studied but little insight to the export processes exists (for review see Okumoto and Pilot, 2011). Despite the lack of understanding of the export mechanisms however, *GLUTAMINE DUMPER1 (GUD1)* overexpression in *Arabidopsis* leads to excess amino acid export. It is not understood exactly how this functions but mutant screens revealed it is the essential component for secretion of amino acids. Yeast-two-hybrid screens and glutathione S-transferase pull-down experiments shows that GUD1 interacts with a RING-type Ub E3 ligase; LOSSOFGUD2 (LOG2). LOG2 is required for the export of amino acids induced by GUD1 (Pratelli et al., 2012). This interaction does not appear to be negatively regulating GUD1 by Ub based degradation but instead may serve as an activation step or perhaps GUD1 and LOG2 form part of a complex for another Ub target which could then act as a repressor or activator of that substrate (Léon and Haguenauer-Tsapis, 2009; Pratelli et al., 2012). Either way this work highlights a significant role for ubiquitination in the homeostasis of nitrogen, via amino acid regulation/secretion.

*NITROGEN LIMITATION ADAPTION (NLA*) mutants in *Arabidopsis* are unable to respond at a transcriptional level to nitrogen limiting conditions. NLA is the only identified component of the plants response to nitrogen limitation and is another RING-type E3 Ub ligase. Mutations within the RING motif of *NLA*, are unable to initiate a response to low nitrogen conditions and they show the same phenotype as low nitrogen grown plants and induce early senescence. NLA is a positive regulator of the plants adaptability to nitrogen limiting conditions (Kant et al., 2011a). This identifies a major role for ubiquitination in the plants response to limited nitrogen and perhaps hints at a universal role for Ub in other areas of metabolism. This claim is reinforced by the fact that two E3 ligases have been shown to play a role in the carbon/nitrogen response (Sato et al., 2009). In addition to this, phosphate homeostasis is in part controlled by NLA in a nitrate dependant manner, and Pi shows an antagonistic cross-talk with nitrate in terms of both their accumulation and influence on the onset of flowering (Kant et al., 2011b). The PHOSPHATE TRANSPORTER1 (PHT1) group of proteins are crucial for Pi homeostasis and these are also regulated by NLA. PHOSPHATE2 (PHO2 also called UBC24), works in harmony with NLA at the plasma membrane, however, data suggest they act independently and both become targets of microRNAs (miR827 and miR399, respectively) under Pi deprivation (Huang et al., 2013). Interestingly PHO2 also induces PHT1 degradation under high Pi (Lin et al., 2013).

The Ub ligases ATL31 and ATL6 have been identified as crucial components of the proper recognition of the balance between carbon and nitrogen during germinative growth. The ratio of carbon to nitrogen is sensed and responded to by *Arabidopsis* and if the peripheral ratio is too high growth is arrested. The *atl31* mutant was found to be insensitive to carbon/nitrogen stress, while the overexpression of *ATL31* resulted in the stress response but under normal conditions (Sato et al., 2009). ATL31 is therefore likely to negatively regulate proteins which detect the ratio of carbon to nitrogen under stress conditions. This underlines the importance ubiquitination has on the various stages of growth and development and again suggests that ubiquitination has a role in a plethora of functions in relation to nutrient homeostasis.

## UBIQUITIN-LIKE PROTEINS AND NUTRIENT STARVATION

In addition to ubiquitination there are other small protein modifiers categorized as Ub-like proteins (Ubls) that can also be conjugated to target proteins. An example of this is the small Ub-related modifier (SUMO). The addition of SUMO to its target can have an effect on its targets ability to bind with the substrate. There are examples of SUMO preventing linkage between the target protein and its binding partner, and also examples of where the SUMOylation enables the binding of the target and its binding partner (for review see Geiss-Fridelander and Melchior, 2007; Miura and Hasegawa, 2010). These mechanisms allow the process of SUMOylation to regulate some of the cells systems by acting as on and/or off switches for rapid responses to various changes in the cell and the environment. SUMOylation has also been linked to the area of nutrient regulation. Phosphate starvation dependant responses have been shown to be under the control of a SUMO E3 ligase; SIZ1 (Miura et al., 2005). SIZ1 controls the activation of the transcription factor PHOSPHATE STARVATION RESPONSE 1(PHR1), which has been shown to bind to the promoter region of the majority of genes which are either up-regulated or down-regulated in response to phosphate starvation (Bustos et al., 2010). Mutant plants with altered SIZ1 show typical phosphate starvation responses when grown on normal conditions, this includes; discontinued primary root development, exaggerated lateral root growth, increased root hair development and excess anthocyanin accumulation, despite the internal phosphate levels being normal (Miura et al., 2005). Further to this deubiquitination has also been shown to play part in the plants response to limited Pi. UBP14 is an Ub-specific protease that acts by modifying root morphology in Pi limited conditions (Li et al., 2010).

Copper is needed by plants in small amounts but is essential for normal development. It is heavily involved in many aspects of growth and development including electron transport, redox reactions and as a cofactor for many metalloproteins. Copper is also toxic is surplus quantities, with excess build-up leading to chlorosis, root growth cessation, and even reduced iron uptake (Burkhead et al., 2009). In high copper conditions, SUMOylation is induced and *SIZ1 *mutant plants grown in excess copper conditions tend to show underdeveloped shoot growth compared to wild-type. *SIZ1 *mutants also accumulate more copper than any other metal relative to normal amounts. Further to this, there is a stark difference in the shoot-to-root ratio of copper in mutant plants compared to wild-type in high copper conditions and accumulation of SUMO1 conjugates is not observed in *SIZ1 *plants whereas it is stimulated by copper in the wild-type (Chen et al., 2011). SIZ1 also regulates nitrogen levels by its E3 SUMO ligase activity. A reduction of nitrate reductase activity is observed in *siz1-2 *plants. Data show this is due to SIZ1 targeting nitrate reductases (NIA1 and NIA2), which become highly active when SUMOylated (Park et al., 2011).

## TOOLS FOR TARGETING UBIQUITIN AND UBIQUITIN-LIKE PROTEINS AND COMPONENTS

Its clear that target identification is key to revealing the influence of UbS in nutrient use efficiency in plants. More sensitive mass spectrometry methods are now available to identify targets for ubiquitination and SUMOylation in planta (Elrouby and Coupland, 2010; Miller et al., 2010). Refined pull-down assays and *in vitro* ubiquitination assays now make finding targets of the process much more straight forward. One method uses detection of thioester bonds between either the E2 and a subclass of E3, or E3 and its substrate (Zhao et al., 2012). This allows for identifying the target protein and the residue to which the thioester bond forms enabling the generation of dominant forms of acute signaling molecules. It can also be used to work back toward identification of the E3 and E2 if the target is already known.

## CONCLUSION

From the evidence here, it is clear that ubiquitination and Ubls play essential roles in uptake, trafficking and maintenance of many of the essential nutrients for plants. However, it is also clear that this area of research in ripe for new discoveries. It is highly likely that understanding the role of Ub in nutrient uptake and processing will provide much needed insight for the development of crops better suited to nutrient deprived land.

## Conflict of Interest Statement

The authors declare that the research was conducted in the absence of any commercial or financial relationships that could be construed as a potential conflict of interest.
